# The Family Environment as a Source for Creating the Dietary Attitudes of Primary School Students—A Focus Group Interview: The Junior-Edu-Żywienie (JEŻ) Project

**DOI:** 10.3390/nu15234930

**Published:** 2023-11-26

**Authors:** Ewa Czarniecka-Skubina, Krystyna Gutkowska, Jadwiga Hamulka

**Affiliations:** 1 Department of Food Gastronomy and Food Hygiene, Institute of Human Nutrition Sciences, Warsaw University of Life Sciences (SGGW-WULS), 166 Nowoursynowska Street, 02-787 Warsaw, Poland; ewa_czarniecka-skubina@sggw.edu.pl; 2 Department of Food Market and Consumer Research, Institute of Human Nutrition Sciences, Warsaw University of Life Sciences (SGGW-WULS), 166 Nowoursynowska Street, 02-787 Warsaw, Poland; krystyna_gutkowska@sggw.edu.pl; 3 Department of Human Nutrition, Institute of Human Nutrition Sciences, Warsaw University of Life Sciences (SGGW-WULS), 166 Nowoursynowska Street, 02-787 Warsaw, Poland

**Keywords:** parents of primary school students, parents’ attitudes, nutritional knowledge, dietary behaviours, focus-group interview (FGI)

## Abstract

The family environment plays a crucial role in creating the health behaviours of children and youth. This study aimed to explore the attitudes of parents with children aged 7–12 who represent an influential environment for creating the eating behaviours of children. A qualitative study was conducted using focus-group interviews (FGI) involving 101 parents from various socioeconomic backgrounds. Three categories of parents were identified based on their level of involvement and awareness of nutrition: ‘aware’, ‘determined’, and ‘relaxed’. Among parents of 10–12-year-old students, an additional category, ‘distanced’ parents, was identified. The study revealed that parents require support in terms of providing compelling arguments and practical recommendations related to meals and reducing or eliminating their children’s consumption of sweets, snacks, fast food, and, in the case of older students, energy drinks. Parents reported that their children had a moderate understanding of the principles of proper nutrition. The majority of respondents viewed this knowledge as primarily theoretical and expressed a need for practical guidance and activities, which they believe should be offered by schools. To achieve positive outcomes in educational activities related to food and nutrition, it is essential to involve children, parents, guardians, teachers, and other school staff in these efforts.

## 1. Introduction

Dietary preferences change throughout life under the influence of biological, social, and environmental factors [1] and are key determinants of dietary choices and, consequently, diet quality [2]. Children’s and youths’ dietary behaviours are derived from the influence of the family and school environment, eating attitudes, and behaviours formed at this age influence diets in later life, including both correct and incorrect dietary attitudes [3,4,5,6,7,8]. The role of the family is particularly important, especially for younger children who have less interpersonal contact with other groups influencing their lifestyles, including eating behaviour. Parents pass on genetic predispositions to their children and create the environment in which gene expression takes place, playing a fundamental role in forming their children’s value system and lifestyle. This role is by definition due to the fact that the family is one of the few examples of a primary group in which, due to, among other things, the direct, so-called face-to-face relationships between members, the internalisation of basic values and social norms takes place.

The family has the greatest influence on children, especially in the first years of their lives, where many quantitative and qualitative changes in their dietary patterns occur, which is closely linked to the child’s development [9]. During adolescence, there is an increasing influence of the peer environment, opinion leaders promoted by social media and the school environment, among others through educational programmes on proper dietary rules [3]. The first stage of adolescence may be the last moment to implement the principles of proper nutrition before relatively stable dietary behaviours and habits are formed [10].

Children model their dietary behaviours, lifestyles, and attitudes related to food consumption and physical activity based on observations of their parents in everyday situations. Children’s behaviours are closer to the ideals of a health-promoting lifestyle the more often parents also exhibit these behaviours [4,6,8,11]. Their eating behaviour is influenced by the availability of different foods, and mealtimes, which provide a natural setting where parents manage children’s behaviour, impose rules and expectations about dietary behaviour, and interact with their children [12,13]. Parents should provide their children with examples of appropriate dietary choices and behaviours that promote physical activity while being positive role models. Therefore, it is to them that educational nutrition programmes should be addressed, taking into account socioeconomic aspects and type of motivation [14,15,16]. The socioeconomic status of the family and especially of the parents plays a significant role in this regard, as it has been proven that more healthy food is consumed in families where parents have a high level of education than in those where parents are less aware of the importance of a proper diet [15].

The mechanisms emphasizing the influence of the family environment on the health behaviours of children and youths are complex [17]. The ability of children to imitate the actions of others and learn through observation, especially from parents and guardians, may explain the types of dietary styles [18,19]. The family is a system where a positive environment can be created that establishes and promotes beneficial health behaviours through role modelling, provision of ‘healthy’ food, and support for engagement in healthy eating behaviours. Appropriate parent practices and communication with the child both can have positive and negative effects on children’s dietary behaviours [18,20].

Previous research has focused on quantitative studies of children’s and adults’ eating behaviours and attitudes. Qualitative research is needed for a more complex understanding of these issues, which will allow, among other things, a better understanding of the substrate of abnormal eating behaviour, which often already arises in childhood. This need is also pointed out by other authors [17,21].

Identifying parents’ concerns and expectations regarding the need for nutritional education is important to determine their level of nutritional awareness, which, in turn, will form the basis for developing the content of educational materials and other messages to support parents/legal guardians in adequately communicating nutritional knowledge to their children.

The aim of the study was to identify the attitudes of parents of primary school students aged 7–12 years towards food and nutrition as an environment that is a source for the formation of children’s and young people’s dietary attitudes.

This aim was accomplished using a qualitative approach because of the following reasons, (i) first, in FGIs, the informative source is a group; (ii) second, the heuristic value of this technique lies in the kind of interaction that emerges during the debate [22]; (iii) third, qualitative research is used increasingly due to its potential to provide meaningful, in-depth insights into participants’ experiences, perspectives, beliefs, and behaviours; and (iv) in addition, the problem of underweight and obesity is a global phenomenon. Therefore, the social context should be sought and the use of FGI may provide additional, significant insight into this complex problem affecting both children and their parents.

## 2. Materials and Methods

### 2.1. Study Design and Participants

Qualitative research was carried out using the focus-group interview (FGI) technique in 16 groups in 10 locations across Poland, varying in size and thus taking into account different socioeconomic aspects. These included metropolitan cities with more than 500,000 inhabitants; cities with 100,000 to 500,000 inhabitants; small towns with up to 50,000 inhabitants; and villages (Table 1). Each focus group was attended by at least 6 parents of primary school students. Parents of children aged 7–9 years (n = 47) and parents of children aged 10–12 years (n = 54) were moderated separately. A total of 101 parents of primary school children aged 7–12 years took part in the study. The research was carried out as a part of the Junior-Edu-Żywienie (JEŻ) project. “JEŻ” is a nationwide project, including quantitative and qualitative research conducted among primary school students, their parents, teachers, and the people responsible for organizing nutrition at school. Based on the research conducted, educational materials were developed for children, their parents, and teachers. Parents from schools that signed up for the JEŻ project were recruited for the study (n = 2218). Teachers from these schools participating in the project recruited parents willing to take part in focus-group interviews. FGIs were conducted separately among parents of students aged 7–9 and parents of students aged 10–12. The children’s parents were similar in age. Most of them were women, and only 10% of participants in the FGIs were men.

### 2.2. Moderation of the FGI

FGI is a qualitative research technique involving in-depth group discussions, typically with 6–8 participants. Individuals are encouraged by a moderator to speak freely and engage in a discussion on specific topics according to a moderation scenario developed in advance and tested in a pilot study [23,24,25,26]. The structure and substantive content are included in the Supplementary Materials.

The study was conducted by three moderators, with one person recording the interviews and taking notes. The interview was explained to the participants before the start. The main moderator initiated the group discussion by introducing the participants to each other. Introduced is the idea of the discussion and the rules governing group behaviour explained (confidentiality, respecting each other’s opinions, and not interfering with each other’s statements). Parents or legal guardians of primary school students aged 7–12 years who gave their agreement to participate in the study were included in the focus group. Among the criteria for exclusion from the focus group were parents of children with health problems (who are on special diets). Participants were able to resign from the FGI at any stage of the study, without giving a reason, but there was no such situation. The issues addressed during the study concerned the problems and challenges parents meet in the context of children’s nutrition, assessing the level of engagement and awareness among parents regarding nutritional matters, and their approach to this area of education. The study also explored parents’ opinions on the current state of children’s nutritional education and the needs in this regard, as well as their ideas on how this education should be conducted.

FGIs among parents were conducted by the specialist from Umbrella Agency Marketing Group. The duration of each group discussion was approximately 90 min. This article discusses topics related to child nutrition problems and the state of knowledge of children aged 7–12 years as perceived by parents.

### 2.3. Procedures and Data Analysis

The group discussions were audio-recorded with the participants’ consent, preserving verbatim statements for the researchers to accurately assess content. Interview transcriptions and moderation notes were coded and initially analysed independently by two researchers. Correlations were examined to reach a consensus.

In the analysis of the results, only the name of the research location was used alongside parents’ statements.

Material from the in-depth group discussions was analysed using principles of grounded theory, focusing on uncovering recurring and prominent themes in participants’ discussions. Data analysis followed a seven-step process: becoming familiar with the data, applying thematic coding, identifying subthemes within the main framework, reviewing and revising the subthemes, defining and naming the subthemes, analyzing and interpreting patterns across the data, and linking the subthemes into dominant contextual domains [26]. During the FGI, the moderators tried to capture the common opinions of the participants, bringing the individual discussion threads to common conclusions, and in case of discrepancies, these different views were noted; parents’ statements recorded by assistant FGI moderators and their generalizations accepted by the respondents or their individual subgroups, as well as the recorded transcripts, were analysed by the authors of the publication who had experience in analyzing qualitative data and described in this text.

### 2.4. Ethical Approval

All procedures involving human subjects received the approval of the Ethics Committee of the Institute of Human Nutrition Sciences of the Warsaw University of Life Sciences (No. 18/2022).

## 3. Results

### 3.1. Recognising Parents’ Attitudes to Food and Nutrition

#### Importance of the Issue and Related Difficulties and Challenges

Parents of primary school students aged 7–9 years.

All parents of 7–9-year-olds participating in the FGI expressed the importance of the topic of good nutrition, diet, and nutritious foods, but also found it to be challenging and unpleasant. This is primarily due to the need for inconvenient, monotonous, and repetitive conversations with children about their food preferences, what they want to eat, and the importance of making healthy choices.

According to some parents who participated in the FGI, children in the 7–9 age group often exhibit reluctance to try new flavours, and their list of favourite foods is relatively limited. Children at this age are still in the process of exploring new tastes, aromas, and textures of various foods, and their palates are not yet fully developed. Additionally, their food preferences may have been created by their experiences in kindergarten, as well as at home with family and grandparents, where different culinary habits, tastes, and food preparation methods may have prevailed. Consequently, children are still adapting to the new culinary habits at school and are just beginning to establish routines for meals away from home, such as having a second breakfast, snacks, or eating in the school canteen. Parents (n = 47) shared during moderations their perceptions and concerns, as well as the daily problems and challenges they encounter in the realm of food and nutrition (Table 2).

It is evident that many of the issues identified are quite common among primary school students aged 7–9 years. What parents of children in this age group need most is inspiration and factual arguments to engage in conversations with their children. The majority of study participants expressed a desire for support and assistance in explaining why certain food groups are either healthy or unhealthy and how they affect their child’s health and proper development. Parents are clearly in need of not only motivation, energy, and patience but also accurate and substantial guidance to effectively implement nutritional education at home.

Of particular concern, however, is the easy accessibility of products that are not recommended for children, not only in local stores but also in school shops. Parents emphasize the challenges in instilling healthy habits in children due to the virtually unlimited variety and availability of snacks and sweets in school shops. According to parents, school shops should inherently promote products recommended for children’s daily nutrition, supplement students’ dietary needs, and support the health-promoting efforts of school canteens. They acknowledge that efforts to promote health among children and adolescents have somewhat reduced access to unhealthy products in schools. However, they believe that school vending machines, present in most of the schools studied, still require monitoring.

As children spend an increasing amount of time at school, the school environment becomes crucial for instilling and reinforcing health-promoting habits. From a young age, children gain a degree of financial independence, as they receive pocket money for their personal expenses. As a result, parents have no full control over their child’s food purchases on school premises and parents would like to trust the institution to ensure that children make health-conscious choices. This involves ensuring that products detrimental to their health are not available in these shops.

Parents of 7–9-year-old primary school students also stressed that they face challenges in terms of good eating behaviours due to modern technology. Inappropriate dietary behaviours are created in children by the media and the internet (websites, social media, internet search engines, etc.). The ubiquitous and intrusive advertisements communicate that a biscuit or colourful cereal is a good choice for breakfast and that a fizzy drink is the best way to quench thirst. In such a situation, it is difficult to explain and convince children to eat, for example, plain oatmeal or nutritious sausage and vegetable sandwiches with water or fruit tea.

Sample statements from parents of primary school children aged 7–9 years:

*‘Children seemingly realise that if they are at home, they will not get sweets before dinner. The condition is to eat dinner. At school it’s out of our hands if they don’t choose a chocolate bar first. A child with money can also buy whatever he or she wants in the grocery shop, and it doesn’t necessarily have to be a healthy product’*.

(parents, Warszawa)

*‘She’s at school and she sees someone, for example, eating something, and then she sees it in a shop. She often asks: will you buy it for me? And you have to come up with something to say no. And the questions arise: why can someone else and I can’t forever?’*.

(parents of children aged 7–9, Kielce)

*‘I don’t understand why vending machines with products such as candy bars, fizzy drinks are so easily available in classes 0–3. For me it is incomprehensible’*.

(parents, Brańszczyk)

The opinions on food and nutrition expressed by parents of primary school students aged 10–12 years (n = 54) are relatively similar to those presented by parents of younger students. They generally agreed that the topic of their children’s nutrition is important. The same as parents of younger children, they are involved in this area of parenting, passing on their knowledge and trying to create correct attitudes. The topic of nutrition education for parents of older children is less problematic, for the following reasons, expressed implicitly in their statements about the behaviour and nutritional knowledge of older children:

1. they have a better understanding of good nutrition and have developed healthy eating habits;
2. they are more open to a variety of food products and are willing to diversify their diets by trying new foods available in school canteens, shops, or at their friends’ homes;
  - they enjoy trying new things and exploring different flavours, even with familiar products;
  - they are often involved in shopping with their parents and have an influence on the shopping list and the contents of the basket;
  - they travel to other countries, where they get to know the local products and dishes and, thus, reduce the level of neophobia;
3. they find the topic of nutrition interesting;
  - they are keen to talk to their peers about the subject, exchange cooking experiences, share products, and try things from each other;
  - they are interested in what others bring to eat and compare it with the contents of their breakfast boxes, aiming to ensure that there are not too many differences, especially if they ‘aspire’ to be group leaders in this respect;
4. they discover the appeal of culinary at home;
  - enjoy spending time in the home kitchen, which is beginning to be attractive to them and is no longer just a place reserved only for adults and associated with urging/forcing them to eat;
  - ask and talk to parents about products, how to prepare meals, and interesting and healthy recipes for different dishes;
5. become independent in the kitchen;
  - they help their parents or prepare interesting dishes/desserts with their own hands, inspired by the example of their peers from the Master Chef Junior programme;
  - they often stay at home without parental supervision and are responsible for preparing a meal for themselves (reheating or even cooking simple food).

The above circumstances influence the attitudes of children aged 10–12 towards nutrition. Parents participating in the study acknowledge that children at this age are less likely to be labelled as “picky eaters”. They are also less selective and more open to trying different foods, which makes their diet more diverse. In general, pupils are capable of defining their taste and culinary preferences. They can distinguish and describe flavours and understand what they do not like and why. In contrast, younger children often reject certain foods on principle, saying “no” simply because they are unfamiliar.

According to the parents taking part in the research (n = 54), other problems arise with 10–12 year olds, more of a social and environmental nature, as primary school students become independent in their extracurricular life, have pocket money, and buy food products themselves. In addition, they spend a lot of time with peers outside the home, without constant parental control. Older children are more active on the internet and create social media accounts, and the internet plays an increasingly important role in their lives. They feel inspired and attempt to create themselves cooking suggestions like the ones they see from Instagram and TikTok influencers. Primary school students, especially those around the age of 12, are gradually entering maturation. Their appearance and physique start to hold greater significance for them. Consequently, it becomes easier to convey messages to them about the influence of nutrition on their bodies, particularly on their appearance, physical wellbeing, and overall behaviour. For example, messages like “fast food leads to obesity”, “diet impacts your skin condition”, or “missing lunch can hinder your performance during training” can be more effective in this age group. All of these factors make them more receptive to more sophisticated arguments compared to students in lower grades. They are also beginning to grasp the consequences of poor nutrition, which is why, according to parents who participated in in-depth group discussions, their children are at a critical age in terms of nutritional education needs. This is the period during which it is still possible to influence and make changes to previously, often unhealthy eating habits. It is also an opportune time to stimulate their interest in food and nutritional issues within the context of public health and the environment and to establish healthy habits that are fundamental for healthcare, as well as for the effectiveness of the learning process, individual wellbeing, and societal wellbeing. Table 3 highlights the challenges and daily issues faced by parents of 10–12-year-old students concerning their children’s nutrition.

Parents of 10–12-year-old students, just like parents of younger children, require strong arguments that effectively communicate to their children why certain groups of products are ‘healthy’ or not. This is because students are increasingly spending time alone, coming home from school without supervision and waiting for other family members to return. Simultaneously, this is the age when they begin to socialize more with their peers. All of this means that parents no longer have strict control over their children’s food choices and decisions. Parents have also emphasized that the availability of snacks in school vending machines poses a challenge to proper nutritional education. In many locations, it is still possible to purchase sweets on school premises.

Sample statements from parents of students aged 10–12:

*‘Legal regulations will not help when the range of vending machines is not controlled. This is where the problem is, vending machines should not have been in schools since 2015, and there is a vending machine full of sweets in our school’*.

(parents, Poręba)

*‘The school should be teaching good habits, but it’s not educating about healthy alternatives. Instead of chips, there should be nuts. Even snacks like dried plums and sultanas are considered sweets’*.

(parents, Kielce)

For older students, the assortment in school shops matters less. The greater concern, compared to younger students, is posed by off-campus shops offering a wide range of products not recommended for children, such as snacks and sweets. Parents emphasize that it is challenging to instill healthy habits in children with such a vast variety and easy access to different types of products.

From an early age, children are spending more and more time away from home, often returning from school on their own, and passing numerous grocery shops, even in small towns. Therefore, parents are aware that education at school and at home must be consistent, setting the same standards and goals, which is a significant problem and challenge.

Examples of parents of pupils 10–12 years old statements:

*‘The children go home by themselves, and there’s a shop on the way. A small amount of money is enough to buy something sweet’*.

(parents, Brańszczyk)

*‘I liked it when there were previous restrictions on sweets and chips in the school shop. Now, everything can be bought again, and as parents, we don’t quite have control. At home, we try to ensure the child eats reasonably healthy. The children have pocket money, and we know they’ll go to the shop and buy whatever they want’*.

(parents, Ostrowiec Świętokrzyski)

*‘For example, my son generally doesn’t eat chips at home, but I know he eats them at school because they’re in the shop. Some products are known to be in the shop, like sandwiches when a child forgets their lunch, but sweet rolls shouldn’t be available’*.

(parents, Kielce)

*‘They are aware that they shouldn’t eat this, but it’s difficult to resist their will. There are so many appealing, colorful sweets in the shops’*.

(parents, Poręba)

It is also worth noting that students of this age are beginning to take an interest in energy drinks, especially boys. In the opinion of many parents, regardless of their place of residence, this is a significant issue and a challenge. This is even more concerning because influential figures, such as internet developers and online gamers, consume these types of drinks. Children lack knowledge and awareness of the potential effects of consuming energy drinks. According to parents, this topic is closely linked to a proper eating style; if children consumed the right number of balanced meals, they would not need stimulant and concentration-enhancing drinks.

Sample statements from parents of 10–12-year-old pupils:

*‘Selling energy drinks to children should be banned, because children don’t know what they are doing and consequently become addicted. Then there are health problems from excess caffeine’*.

(parents, Białystok)

*‘There is a problem with the energisers. I don’t know if this problem is raised in school. There shouldn’t be such easy access to them at all. A lot of children of an age like my son, around 12, and even younger already try these drinks. And that’s what’s dangerous, because the body of such young people reacts differently. I try to make my child aware that it’s not like water, and drinking energy drinks is a health hazard’*.

(parents, Lublin)

### 3.2. Typology of Parents Participating in the FGIs in Terms of Involvement and Approach to Food and Nutrition

Within the study’s participants, a differential typology can be established based on the level of engagement and awareness of nutrition-related issues, as well as their approach to this aspect of parenting and education. Among parents of primary school children, three profiles were identified: “aware”, “determined”, and “relaxed”. In the case of 10–12-year-old students, an additional profile of “distanced” parents was recognized. Their characteristics are presented in Table 4. It is noteworthy that the subject of proper nutrition for students aged 7–12 years was deemed significant by all participating parents in the study.

#### 3.2.1. Parent Profile “Aware”

Parents representing the ‘aware’ profile (n = 16) constitute the smallest group and are more likely to be women residing in a city and in larger rural areas near major urban centres.

Parents who fall into the “aware” profile exhibit distinctive characteristics in their approach to food and nutrition. They pay close attention to and are concerned about the dietary habits of the entire family, not just their children. They take pleasure in and have a genuine interest in topics related to nutrition and its impact on health and the body. They adhere to the mantras *‘I know what I eat*’ and *‘you are what you eat’*.

Their concern for what they consume arises from the satisfaction and pleasure it brings them, along with the clear positive effects on their wellbeing, physical and mental health, and overall health. Some of them have maintained a proper diet throughout their lives, while, for others, the change was triggered by specific events, such as illness, unfavourable test results, obesity, declining health, or the sudden loss of a loved one. They are well-informed about the alternative market options and can find high-quality, local products with a “clean label” even in popular discount stores and supermarkets. Despite their strict dietary preferences and eating habits, this comes naturally to them, as they feel comfortable maintaining a healthy dietary routine.

It is evident that children of parents in this profile encounter fewer challenges regarding nutrition and adhering to dietary restrictions. The children are accustomed to consuming vegetables, fruits, legumes, fish, and whole grain products, which form the foundation of their diet.

Parents belonging to the “aware” profile often engage their children in discussions about nutrition and take an interest in this area of knowledge. In their households, it is a significant and engaging subject. The children’s nutritional education naturally unfolds during grocery shopping and meal preparation. Good eating habits are passed down from parents to children. The entire family follows consistent dietary guidelines and is well-informed about proper nutrition. They often hold views such as plant-based milk being superior to cow’s milk and the belief that organic certification is the sole indicator of quality, among other things.

Sample statements from parents of pupils 7–9 years old:

*‘As the child grows older, awareness grows, but from a young age it is necessary to instil proper dietary rules. Teach children how to follow the rules of proper nutrition’*.

(parents, Kielce)

*‘Good arguments are needed. You want to live longer, eat healthy. You want to be pretty eat healthy. You want to have nice skin, eat healthy. These are the kind of arguments I try to pass on to my child all the time’*.

(parents, Nowy Sącz)

Sample statements from parents of pupils 10–12 years old:

*‘My child is exposed to healthy products from a young age. For him, eating vegetables is something natural’*.

(parents, Warszawa)

*‘It’s just that if we eat wholemeal pasta, we all do. My child doesn’t know any different and he doesn’t have a choice’*.

(parents, Czachówek)

#### 3.2.2. Parent Profile ’Determined”

This group of parents, who have children in both the 7–9 (n = 30) and 10–12 (n = 25) age groups, is the most numerous (Table 4). In the case of parents with children aged 7–9, it comprises both men and women from rural and urban areas, with the largest group residing in larger cities. For parents of 10–12-year-old students, it includes men and women from various locations, with a predominance of larger and medium-sized cities.

These parents make a concerted effort to instil healthy eating habits in their children, but, at the same time, they find it challenging. They are aware of the importance of being role models in this regard, although it is not always easy to achieve. They care about and recognize the value of promoting good eating behaviours within their households, particularly among their children.

Parents of younger children struggle to maintain specific restrictions and healthy habits, even though they desire to do so. They employ various methods to encourage a healthy eating style at home, often resorting to directives (e.g., setting a portion of vegetables for lunch) and prohibitions (e.g., no sweets from Monday to Friday) as a means of education. They occasionally seek out recipes for “healthy” dishes on their own initiative or modify their favourite meals to make them both delicious and beneficial to their health (e.g., using whole-grain flour instead of regular flour). However, in their daily lives, they may feel uncertain about diversifying and planning their meals. They typically shop for groceries based on a standard list and may struggle to identify valuable products. Still, if they come across recommendations or someone suggests healthier options, they are willing to try and make changes.

Parents of 7–9 year olds with a “determined” profile devote a significant amount of time and attention to nutrition principles. However, it is essential to highlight that parents are often more interested in the topic than their children. Their primary goal is to engage their children in learning about and following proper nutrition principles, which can be challenging. These parents may lack nutritional knowledge but are reluctant to admit it.

Parents of 10–12 year olds differ from parents of 7–9 year olds in having a more relaxed approach to the topic of nutrition. Parents of younger children tend to feel more tension and stress regarding nutrition. Nevertheless, both groups are equally committed to ensuring their children adopt healthy eating habits and strive to meet these expectations.

Children aged 7–9 were continuously supervised by parents, grandparents, or caregivers at school or daycare, who determined meal times and types. In contrast, 10–12 year olds are entering a stage of independence and responsibility for their actions and food choices. They decide what to eat when they are home alone after school, and if they do not like their school lunch, they may choose not to eat it and may even spend their pocket money on snacks or sweets. As a result, parents of older “determined” students are aware that they can no longer control all their children’s behaviours.

Some parents in this group of older students are deeply concerned that their children understand the consequences of their dietary choices, including the positive and negative effects on their bodies, health, behaviour, and emotions. They believe this is the last chance to instil the right attitudes towards food and nutrition, which will ultimately lead to changes in their habits and bear fruit in the future.

Examples of parents’ statements:

*‘When shopping ‘on the go’ I don’t read labels. I take what is on the shelf. I don’t have time to read the label’*.

(parents of pupils aged 7–9, Kielce)

*‘We try to make sure we eat healthy and stay healthy. We try, but unfortunately there is resistance from some of the household members, so it doesn’t always work out, but we try’*.

(parents of pupils aged 7–9 years, Nowy Sącz)

*‘The food pyramid alone is not enough, because children know what the nutrition basis is. It’s all explained in grade 1 or 2, but experiential learning is important. Talking about what problems a person has when they are obese, i.e., that they have heart disease, high blood pressure or diabetes, can make children more aware of the scale of the problem’*.

(parents of pupils aged 10–12, Lublin)

*‘My children watched the film ‘Super Size Me’ about a man who ate at McDonalds for 30 days. It made an electrifying impression on them’*.

(parents of pupils 10–12 years old, Białystok)

*‘They know that what is unhealthy, but they don’t know what it can be, what the consequences and harm can be. For example, diabetes, but they don’t know what the disease entails. Children think that food cannot be harmful’*.

(parents of pupils 10–12 years old, Ostrowiec Świętokrzyski, Poręba)

#### 3.2.3. Parents Profile “Relaxed”

Parents falling into the ‘relaxed’ profile (n = 22), whether with younger or older primary school students, are often men residing in smaller towns or rural areas. They recognize the importance of proper nutrition but feel it is a luxury that only those with ample free time can afford. They argue that, given the numerous responsibilities parents and children have today, maintaining a healthy diet is challenging. They contend that children are generally active, both at school and during play, which they believe compensates for any shortcomings in their diet. These parents admit to having limited nutritional knowledge. They are familiar with the basics, but they do not see the need to expand their knowledge on the subject. In their view, schools should be responsible for providing children with the most critical nutritional information. Some believe that rational nutrition should not be a heavily emphasized subject. They adhere to the principles: *‘food is necessary for life, so you have to eat something’*, *‘everything is for people after all’*.

For parents within this profile, it is important to maintain a minimum level of nutrition for their children. This includes ensuring that the child has lunch during the day, preferably at school, prepares a sandwich for a second breakfast, consumes vegetables and fruit several times a week, and limits sweets, though without strict boundaries. Parents with a “relaxed” profile are the least informed and reluctant to discuss dietary guidelines with their children. They tend to be quite apathetic about the subject, sometimes even following the ‘*nothing by force*’ principle. This group of parents generally lacks adequate nutritional knowledge. Among the parents who participated in the study, they are the most likely to overlook issues like excessive sweets consumption and a shortage of vegetables in their children’s diet, among other things.

Sample statements from parents:

*‘You cannot eliminate sweets 100 percent. You can only introduce moderation. I am of the opinion not to overdo it in the other direction’*.

(parents of pupils aged 7–9 years, Kielce)

*‘I for one am not in favour of us not giving sweets to children because it is a pleasure for them. At our house we have a rule ‘eat the fruit first then you will get some sweets’. I can’t completely deprive children of, for example, chocolate, they can probably have a bit of pleasure’*.

(parents of pupils aged 7–9, Nowy Sącz)

*‘On a day-to-day basis, the biggest problem I have when it comes to feeding my child is with time. I just don’t, I have time to deal with this subject’*.

(parents of pupils 10–12 year old, Białystok)

*‘At my home sweets are generally available, my son takes when he wants’*.

(parents of pupils 10–12 years old, Kielce)

*‘We all like it. When I buy chocolate, there are four of us, everyone has their own. In the same way, everyone has their bottle of cola’*.

(parents of pupils 10–12 years old, Kielce)

*‘Sometimes a person is only human and has a craving for something unhealthy. Everything is for people’*.

(parents of pupils 10–12 years old, Ostrowiec Świętokrzyski)

#### 3.2.4. Parents Profile “Distanced”

The ‘distanced’ parent profile (n = 8) is specific to parents of primary school students aged 10–12. As these primary school students have a more extensive curriculum and start learning various new subjects, such as biology, mathematics, and history, grades and knowledge become increasingly important. Consequently, some parents of older pupils no longer consider the acquisition of nutritional knowledge as a priority. This group is more likely to consist mainly of women from large cities who are specialists in their respective fields, prioritizing their busy careers. They believe that children at school should primarily focus on acquiring scientific knowledge to ensure a good education. In their perception, nutritional education is not knowledge but rather a matter of habits and customs that can be easily acquired.

Sample statements from parents of pupils 10–12 years old:

*‘You simply have to eat. However, there are more important things, such as learning’*.

(parents, Warszawa)

*‘It is only important that food choices are not random’*.

(parents, Ostrowiec Świętokrzyski)

### 3.3. Current Nutritional Knowledge of Children as Perceived by Parents

The majority of parents (n = 91) assess their children’s nutritional knowledge as reasonably good, even though they perceive it as being too theoretical and not practical enough. Parents believe that their children primarily receive information about food and nutrition at school and, before that, in kindergarten. According to parents, their children understand why they should avoid consuming products from certain food groups, but they are less aware of the risks these products pose to their health. In cases where they do recognize the risks, they typically identify only the basic ones, such as the risk of obesity associated with excessive sugar consumption. Another significant aspect is the lack of awareness regarding potential long-term side effects resulting from an unsuitable diet. The same applies to the food pyramid, which serves as the foundation of the curriculum and marks the beginning of children’s nutritional education. However, children tend to be more familiar with its theoretical dimension than its practical application.

Knowledge about food products and food production methods is rated slightly lower by parents. They believe that these areas receive less emphasis in the curriculum. Parents residing in large cities emphasize the importance of their children acquiring this knowledge to dispel the misconception that supermarkets are the primary source of food. In our study, practical knowledge in this regard is more likely to be gained by 7–9-year-old students from smaller towns or rural areas who have direct exposure to vegetable gardens, agricultural fields, or animal husbandry for meat production.

Parents participating in the study note that the various activities organised by the school to promote healthy eating, e.g., *Healthy Eating Day, Red Vegetable Day*, *Yellow Fruit Day,* etc., have a positive impact on their children. In some of the schools participating in project JEŻ (Junior-Edu-Żywienie), e.g., Nowy Sącz, Warszawa, and Czachówek, the school organized practical activities consisting of preparing meals together, e.g., salads, fruit smoothies, or even breakfast, although such activities are, in their opinion, too rare. On the other hand, permanent campaigns organised by teachers, such as ‘you get points for a vegetable in your breakfast tray’, carried out, for example in Brańszczyk or Ostrowiec Świętokrzyski, are not recognised by parents as promoting healthy eating habits. This is because children are rewarded if they have brought in, but not necessarily eaten, healthy products.

Sample statements from parents of 7–9-year-old pupils:

*‘It’s nicely packaged. It says eat me. The adverts about sweets, crisps and all that kind of snacking, that’s what the child fights for. He sees it and that’s what he wants afterwards’*.

(Nowy Sącz)

During FGIs, parents of 10–12-year-old primary school students, like younger students, rate their children’s knowledge of the principles of good nutrition highly, although, again, they consider this knowledge to be theoretical. Older children regard nutrition education—e.g., in biology—as knowledge to be learnt and passed for marks. They lack the ability to translate what they have learned into practice, which may be a recommendation for teachers to introduce a more practical thread to these lessons.

In the opinion of the participating parents, there is a need for activities that make pupils aware of the purpose of learning a rational diet from the early years of school and even kindergarten.

Sample statements from parents of students aged 10–12:

*‘You are told in such vague terms what is healthy, this has this vitamin and that has another vitamin. This is good for you and this is bad for you. Just theorising is of little use. It’s more like if you eat this product, you’ll have a lot of energy. If you eat this product, you won’t have energy. We adults already know that if we eat a tuna sandwich with, for example, lettuce, we’ll feel great, and if we eat a bowl of pop-corn, we won’t feel good’*.

(parents, Białystok)

*‘It seems that a practical approach to the subject is important. Only theoretical knowledge will not be effective’*.

(parents, Białystok)

*‘Myself, I think drastic examples would work, what happens when you consume products that are not recommended and what happens when they are recommended. What effect it has on appearance, illnesses. Fear is a strong stimulus’*.

(parents, Poręba)

Based on group interviews, it can be concluded that, as in the case of pupils aged 7–9, older children also lack an idea of the consequences of poor nutrition far into the future. Parents during the study shared examples that their children constantly reproduce patterns and incorrect habits. They are unable to find solutions on their own, despite having sufficient knowledge.

Here is what one mother of a student 10–12 year old said about the lack of nutritional recovery after exercise and too long of breaks between meals: *‘When my daughter comes back from training and is tired, she doesn’t want to eat and after an hour she starts to get a headache. Even though I explain to her: you come back, you absolutely have to eat, only then can you do other things. And this happens once, then a second time and again. The third time she comes to me and says: I did it again, I have a headache, I won’t do it again. Children have to practice bad and good behaviour with themselves. Experience it for themselves. To see what it really means’* (mother, Białystok).

According to parents of 10–12-year-old students, children lack knowledge about food production. These topics appear particularly unfamiliar and distant in large cities. However, students are genuinely interested in learning how various products are manufactured. They eagerly watch programs like *‘How it’s made’* or *‘Galileo’*. Therefore, it seems that this age is appropriate for making students aware of the processes involved in food production. There is a good chance that they will remember this knowledge well and, more importantly, apply it in practical situations, such as when shopping on their own.

## 4. Discussion

### 4.1. Parents’ Nutritional Problems and Challenges for Children Aged 7–12 Years

Parents (n = 47) participating in the focus group interviews pointed out that, for children aged 7–9 years, a limitation to the introduction of an appropriate eating behaviour is the fact that they prefer, for taste reasons, a limited number of foods and are consequently characterised by a selective choice of foods in a diet that is not varied. In contrast, pupils aged 10–12 are already more aware that nutrition plays an important role in maintaining health. However, the emerging influence of the peer environment, as well as traditional and social media, results in the consumption of products that are not recommended and categorised as junk food, as well as those not allowed to be consumed at this age, such as energy drinks. These behaviours depend on the nutritional awareness of parents, who have a huge influence on children and adolescents at this age. The social influence on children aged 7–9 manifests itself in imitation and automatic acceptance of the behavioural reactions of those around them, as well as in obedience to the authority of parents. Adolescents aged 10–12, on the other hand, are more influenced by the peer group with which they identify and emulate.

As research has shown, parents need support in this area, understanding the role of proper nutrition in maintaining health. Indeed, proper nutrition is an essential factor for the harmonious development of the young organism and the attainment of high health potential, as well as shaping appropriate eating patterns in adulthood. Early modifications of eating habits, especially in childhood, can promote health and reduce the risk of developing diseases in later life.

Eating regular main meals and refraining from snacking between them, particularly on foods with low nutritional value, plays a crucial role in the proper utilization of nutrients provided by food. Snacking on such items increases the risk of being overweight and obese. Conversely, skipping main meals can lead to nutritional deficiencies, which can be a contributing factor to various diet-related diseases [8].

A meta-analysis of 57 studies involving 203,706 participants revealed that shared family meals have a significant impact on children’s eating behaviour [27]. The internalization of appropriate eating behaviours, often referred to as ‘food socialization,’ should commence with shared meals at home, fostering a positive atmosphere during mealtimes, and actively teaching a health-promoting lifestyle [28]. Within the family context, it is crucial to instil proper eating habits, make informed food choices, and maintain the frequency and regularity of family meals. These factors positively influence the eating behaviours of children and adolescents [29,30] and are inversely related to the prevalence of being overweight [31,32]. Eating meals with family is associated with the development of healthy eating patterns and improved diet quality [33,34,35,36]. It is linked to a higher consumption of fruits and vegetables, fibre-rich foods, and foods rich in micronutrients, calcium, and vitamins. Simultaneously, it is associated with reduced consumption of fried foods, fast food, and foods high in saturated fats, trans fatty acids and simple sugars [8,27,34,37]. Adolescents and children who infrequently participate in family meals tend to consume more unhealthy foods. It is crucial to emphasize that the positive impact of family meals hinges on the type of food served. Purchasing certain takeout foods (e.g., fast food) and consuming them during family meals can counteract the benefits associated with home-based family meals [37,38]. Research findings indicate that only 50.7 percent of 9-year-olds have family meals, and, among 14-year-olds, the percentage is 35.4 percent [27,39].

The benefits of family meals extend into adolescence and early adulthood. Young adults who had daily meals with their families during adolescence consumed more fruits and vegetables compared to their peers who did not have this practice at home [40]. Among these individuals, eating disorders are less common [41], and healthy eating patterns are more likely to be established [42]. Promoting family meals as a strategy for developing and maintaining healthy dietary habits in children has proven to be effective. Moreover, a study found that eating four meals a day, as opposed to three, is associated with a reduced risk of developing type 2 diabetes [43].

Breakfast is considered the most important meal of the day [32]. However, researchers [44,45,46,47] estimate that it is not regularly consumed, with a significant proportion of students going to school without this meal. This practice can lead to fluctuations in blood glucose levels, affecting the ability to concentrate, limiting cognitive processes, and resulting in poorer academic performance. Moreover, the hunger experienced when skipping breakfast is often satisfied with sweets and snacks available in school shops. These irregularities may be responsible for changes in metabolic rates [48]. The frequency of family breakfasts is linked to various dietary factors, including a higher intake of fruits, whole grains, and fibre, as well as a reduced risk of being overweight or obese [47]. Having breakfast as a family and sharing dinner in the evening positively influences eating behaviour, including making appropriate food choices in the diet [27,49,50]. It also promotes children’s mental health [51]. As the frequency of family mealtimes increases, so does the sense of self-efficacy for healthy eating, which, in turn, encourages healthy eating behaviours [52,53].

Parental practices exert a significant influence on children’s nutrition, affecting both food choices and meal regularity. When parents follow a restricted diet, certain foods may be absent from the family table, potentially contributing to neophobia among children and adolescents, particularly towards healthier but unfamiliar food alternatives [54].

To shape children’s food preferences and behaviours, parents employ a range of methods that combine effective and ineffective forms of influence, including overt and covert control strategies [15,55]. Overt control involves implementing various restrictions and limitations on food consumption, as well as pressuring children to eat certain foods or meals, especially when dealing with those who are resistant to eating (‘picky-eaters’). On the other hand, covert control entails strategies, such as purchasing only healthy foods and avoiding stores and restaurants that offer ‘unhealthy foods’. Children may be aware of overt control but may not recognize covert control [15].

It is important to note that the use of restrictive dietary practices by parents can be counterproductive and lead to increased consumption of restricted foods, potentially increasing the risk of excessive weight gain [19,56]. However, the same authors [56] emphasize the necessity of parental control in limiting children’s intake of foods that are not recommended. Some researchers suggest that excessive parental control should be avoided, as it may impede a child’s development of self-regulated feeding practices, including recognizing hunger and satiety signals [57]. The parenting practices that have been most positively associated with children’s eating behaviour are role modelling, parental encouragement, and moderate restriction [58,59].

Another parental practice involves using foods as rewards, particularly those that are high in fat and sugar. Numerous studies have demonstrated that repeatedly offering snacks or sweets as rewards can lead to an increased preference for such foods [56] while decreasing the preference for foods promoted as health promoting [60,61]. This behaviour is often exhibited by parents who align with the ‘relaxed’ or ‘distanced’ profile, in which the belief that ‘*everything is for the people*’ prevails. Some parents (n = 32) in our study also argue that it is not feasible to eliminate sweets from their children’s diets, as prohibiting them may only make the children desire these items more. Furthermore, they believe that it is impossible to protect children from sweets, as they are bound to receive them as gifts from grandparents, friends, or during peer birthday parties sooner or later.

Parents consider family meals as an opportunity to enhance interaction with their children and convey their values regarding food and nutrition. However, the act of watching television during family meals seems to negate the associated benefits [41]. This is due to the tendency to overeat while watching television and the potential for children to learn eating habits from commercials and other programming [41,62]. Children influenced by advertising are at an increased risk of being drawn to low-nutrition products that are heavily promoted, as demonstrated by a study in New Zealand [30]. This susceptibility is rooted in the characteristic features of children and adolescents as advertising audiences; they often lack critical thinking skills, are gullible, and perceive the message in an emotional and literal manner, without fully understanding its essence or the persuasive nature of advertising. Currently, children spend approximately 4–5 h per day engaging with various forms of media. In many cases, this constitutes a significant portion of their leisure time. Even the youngest preschool children spend more time in front of a TV or computer screen than playing outdoors in their backyard [63,64,65,66].

At the age of 10–12 years, in preadolescence, there are tendencies to manifest one’s independence, which manifests itself in rebellion against parents and other authority figures, while, at the same time, the need for acceptance by peers emerges. The peer group plays a key role during this period [67,68]. Young people are susceptible to negative environmental patterns and readily adopt unhealthy behaviours [69]. During this period, the independence of adolescents increases; they spend a lot of time away from home, also using various catering establishments, so parents have less control over what they eat, which results in the modification of eating behaviour patterns brought from the family home. Teenagers make their own food choices, which are not always appropriate and are easily influenced by advertising, fashion, and patterns created by their peers. There is a significant level of food neophobia in students of this age, characterised, among other things, by a lower intake of vegetables [70].

In the present study, parents indicated a problem with the consumption of snacks, fast food, sweetened drinks, or energy drinks (n = 95). In this respect, they expect support from the school, which does not always create positive role models by locating vending machines with inadvisable sweets or drinks on school premises. At the same time, they themselves, as also pointed out by other authors [71], give money to children, knowing that it will be used mainly to buy snacks, candy bars, crisps, cakes, cola, fast food in school tuck shops, vending machines, or shops on the way to and from school [72]

### 4.2. Pupils’ State of Knowledge as Perceived by Parents

Nutrition knowledge is widely acknowledged as an integral component of healthy eating behaviour [73]. Parents who participated in this study emphasized that both 7–9-year-old (n = 47) and 10–12-year-old (n = 54) pupils possessed relatively good knowledge of nutrition but lacked practical knowledge of food. From an early age, educational institutions incorporate elements of a healthy lifestyle, but it has been noted that this knowledge is primarily theoretical. Practical knowledge in this area is deemed necessary, as emphasized by other authors as well [74]. In another studies [30,75,76], the authors found that children can correctly identify foods that are either beneficial or unfavourable to their health but struggle to apply this knowledge in their daily behaviour. Children’s food knowledge, preferences, and, ultimately, their food consumption are closely intertwined with their parents’ preferences, beliefs, and attitudes towards food. This linkage arises from the availability of products that parents like, purchase, and consume. Therefore, the role of parents in fostering healthy eating habits is crucial, as underlined in this study. Nevertheless, parents have pointed out the need for substantive and practical support in this area, as they sometimes struggle with certain issues in this regard.

### 4.3. Parents’ Needs in Terms of Children’s Nutrition Education

The accelerating pace of life, insufficient financial resources, long working hours, and at times, a lack of motivation, mean that some parents do not have the time, opportunity, or inclination to prioritize the quality of their children’s diet. This leads to irregular eating patterns, frequent snacking between meals, and the consumption of non-nutritious foods and meals, all of which have a negative impact on health. Inadequate education regarding the principles of healthy eating and limited access to nutritious food options to replace high-calorie snacks consumed between meals contribute to the proliferation and persistence of unhealthy eating preferences and behaviours.

The consistent lack of time among parents results in a transfer of responsibility for educating their children about health and nutrition to the school. All parents who participated in the TGI expressed a need for nutritional education for their children at school, along with educational materials designed for them.

Appropriate nutrition education for both children and their parents/legal guardians and teachers can support the formation of eating habits in a health-promoting direction [5]. It appears that collaboration between the family environment and the school can have a more effective impact on children’s eating behaviours than individual actions, as also pointed out by other authors [77,78,79].

The eating behaviours of children and adolescents are a product of the influence of two social environments: the home/family environment and the school environment. Many authors emphasise the need for multilevel education from kindergarten through school, involving children and teachers, the media, and parents, and up to medical personnel [80,81].

A high level of knowledge of children and adolescents about healthy eating is a factor in shaping health-promoting eating behaviours. Many authors [63,78] find a positive relationship between knowledge and correct eating behaviour and the perpetuation of health-promoting behaviours.

During their time in school facilities, children are exposed to various influences related to values and opinions, including those concerning food and nutrition. Through the provision of nutritional knowledge in the classroom and the meals served in the school cafeteria, the eating behaviours of school children and adolescents can be moulded. The goal of nutrition education is to instil an understanding of the principles of proper nutrition and cultivate the skills, beliefs, and attitudes necessary to maintain and enhance one’s own health and that of others [82,83,84]. The scope of this education should encompass the transmission of knowledge regarding the principles of balanced nutrition, the development of attitudes toward various diets (e.g., vegetarian or low-calorie diets), the cultivation of menu planning skills, the preparation of food to preserve its nutritional value, the selection of food products (including the habit of reading product labels), proper food storage, aesthetically arranging the dining table, and creating a pleasant atmosphere during meals.

To sum up, the qualitative research was conducted using the FGI method among parents of students in grades 1–6; it should be noted that there is a necessary synergy of interactions between parents and teachers in shaping the nutritional attitudes of their children. In order to convey common educational content, during regular meetings between educators and parents of students attending these classes, we should talk about proper nutrition and its role in achieving students’ physical fitness and cognitive function, as well as in the prevention of diet-related diseases. It is also worth proposing joint practical activities involving games and gamification, in which not only teachers but also students could be encouraged to participate.

The implementation of these recommended activities will also be supported by educational materials developed as part of the “JEŻ” project, especially a book containing rebuses, comics, and crosswords that can be solved by students with their parents, as well as a cookbook with suggested recipes for dishes that can also be made by children.

## 5. Conclusions

Based on the research findings, it has been determined that parents require support in addressing food and nutrition issues, including providing sound arguments and practical recommendations to help instil healthy habits and attitudes in their children. The goal is to empower children to make informed and health-conscious food choices. Parents of children aged 7–9 years often struggle with convincing their children to consume a variety of foods regularly and to reduce their consumption of sweets. Parents of 10–12 year olds frequently face challenges related to restricting their children’s intake of sweets, snacks, fast food, and energy drinks.

Addressing the behaviour of school-age children necessitates the development of intervention proposals that consider the complexity of their attitudes and behaviours regarding food and nutrition, as well as their emotional and nutritional requirements. Understanding the perceptions and significance of good nutrition among school-age children can inform strategies aimed at making nutritious foods more accessible, attractive, and normalized within their peer groups. Furthermore, it is apparent that parents may lack creativity in diversifying meals, emphasizing the need to create and promote simple recipes for preparing various meals at home with both parents and children, as well as with peers.

Effective food and nutrition education initiatives should engage children, adolescents, parents or legal guardians, teachers, and other school staff, as only multilevel approaches can produce positive effects.

## Figures and Tables

**Table 1:** Residence characteristics of parents participating in the research.

Type of Locality	Size of Locality	Location
village	330–2000 inhabitants	Rosko, Czachówek, Poręba
city	up to 50,000 inhabitants	Brańszczyk
city	100,000–500,000 inhabitants	Białystok, Lublin, Kielce, Ostrowiec Świętokrzyski, Nowy Sącz
city	over 500,000 inhabitants	Warszawa

**Table 2:** Parental concerns and challenges related to nutrition of children aged 7–9 years.

Problem	Description of the Problem	Parents’ Needs
Reluctance of the child to eat meals (so-called “non-eater” children)	students’ diet is limited and unvaried, with small diversification of the product; they selectively consume fruits and vegetables	Information about the ways of expanding the diet with new products; enriching meals with products rich in vitamins, minerals, and nutrients; to ensure a variety of products and thus a diversity of nutrients in their diet
The child’s reluctance to eat healthy and nutritious foods, i.e., vegetables, fish, cereal products	lack of understanding and knowledge among children about the functions of individual nutrients and their benefits for the body; why they support their development	age-appropriate examples and arguments (mainly practical rather than theoretical) to influence children’s attitudes towards eating healthy and nutritious meals
Contents of the breakfast box	lack of ideas and creativity on a daily basis; the child’s second breakfast is monotonous and duplicated every day, but parents prefer to give a tried and tested set of foods that the child will eat and not throw away	inspiration for the creation of tasty, but optimal meals, with the right energy value, containing vegetables and fruit; ideas needed for quick and simple, tasty, and nutritious sets for a second breakfast
Why and what to eat for the first breakfast	students skipping breakfast or eating inappropriate products	arguments to convince the child why this meal should be eaten; the role of breakfast and the choice of foods that give the most benefit to the body in the morning
Independent choices when shopping for groceries	bad habits of students with buying a snack and drinks from vending machines on school premises and in shops in/outside the school	how to develop good habits and attitudes in children so that they make conscious food choices that are good for their health
The role of different meals in the diet	students do not eat breakfast, lunch, or dinner	how to convince your child to eat lunch at school, how to explain that these are important meals for their health and what are the effects and risks of skipping them; how meals should be composed/balanced and why they should not be replaced by sweets or snacks
Allowed amount of sweets/sugar	students’ excessive consumption of sugar and sweets in their diet	how to explain to your child that sweets are harmful; what is the optimum amount of sugars per day/week; which sweets to allow your child (i.e., choosing the “lesser of two evils”) and in what quantities

**Table 3:** Parents’ problems and challenges related to nutrition of children aged 10–12 years old.

Name of the Problem	Description of the Problem	Parents’ Needs
What constitutes good physical activity	Pupils avoid physical activity and prefer a sedentary lifestyle.	The difficulty of explaining to a child the role of sport in life, especially in the process of growing up. How to encourage your child to exercise; how to eliminate e-sports instead of real physical activity
Emotional eating	Difficulties coping with different emotional situations of pupils, e.g., stress, boredom, euphoria, or anger	Support for rational explanation of the circumstances under which different emotional states arise and knowledge of the mechanisms of rationalisation and consequently coping with these emotional states
Disadvantages of fast food	Students consume too much fast food, often without the parent’s knowledge	Support for the argumentation of the need to eliminate or significantly reduce the consumption of “junk food”; by, inter alia, explaining what changes in the body and health consequences they cause.
Child’s reluctance to eat healthy and nutritious foods (e.g., vegetables, fish, and cereal products)	Students avoid eating valuable food products such as fish, cereals, and vegetables, choosing only those they like	Support to persuade children to choose health-promoting foods, examples of which children are familiar with but rarely interested in consuming due to, among other things, a lack of understanding among children of the benefits of these products for the body, their functions and why they support their development. Proposals for age-appropriate examples and arguments (more practical than theoretical) to influence children’s attitudes towards eating healthy and nutritious meals
Why you should eat breakfast and what you should eat for breakfast	Students skipping breakfast	The role of the meal itself and the choice of foods that, when eaten in the morning, give the most benefit to the body. Examples of arguments convincing children of the necessity of eating first breakfast
Independent choices in grocery shopping	Independent purchases of snacks and carbonated, sweetened drinks by students	What to buy and what to avoid in the snack vending machines located in the school and in shops in and outside the school. A proposal for teaching children the right habits and attitudes to choose the right products for their health
The role of different meals in the diet	Students skip meals such as breakfast, second breakfast, lunch, and dinner in their diet.	Support for explaining the importance of different meals in the diet for their health and what are the effects and risks of skipping them; support for examples of composing meals without sweets and snacks. Why it is important to eat breakfast, lunch, and dinner regularly; how to get your child to eat lunch at school
The antinutritive nature of sweets	students’ excessive consumption of sugar and sweets in their diet	Supporting the explanation of the antinutritional and antihealth benefits of consuming excessive amounts of sweets. Ability to determine the optimum amount of sugars per day/week and justify it
Adverse nature of energy drinks	Students experiment with energy drinks, encouraged by advertisements	Support for the ability to demonstrate the adverse health effects of energy drinks. Reasons not to recommend the consumption of energy drinks by children

**Table 4:** Typology of parents of students aged 7–12 years based on their level of involvement and awareness of nutrition.

**Profiles of parents of children aged 7–9 years (n = 47)**			
“aware” n = 7	“determined” n = 30	“relaxed” n = 10	--
**Profiles of parents of children aged 10–12 years (n = 54)**			
“aware” n = 9	“determined” n = 25	“relaxed” n = 12	“distanced” n = 8 *
**Involvement of parents in food and nutrition topics:**			
high level	high level	low level	intermediate level
**Parents’ approach to nutrition:**			
eat rationally (lots of fruit and vegetables, optimum number of meals a day and breaks between them); avoid processed products (preservatives, colourings); read labels, are “familiar” with the ingredients, look for, e.g., bio and eco certificates, fair trade, and country of production, etc.; are often vegetarians/vegans; appreciate the quality and local nature of products, e.g., they cultivate home gardens, do their shopping at local markets, play sports, take care about physical activity with the whole family; they like to cook, experiment and fulfil themselves in the kitchen (they prepare homemade hummus and vegetable pizza); they practically do not eat out; they do not have “standard” sweets in their homes, they buy sweets based on natural sweeteners (erythrol, xylitol); a few of them belong to food cooperatives and community gardens (parents of children aged 7–9 from big cities)	they try and would like to eat healthily and rationally; they are aware that nutrition has an impact on quality of life and health; they sometimes read labels when they are not sure about a product, but their knowledge of ingredients is very basic, they avoid e.g., glucose–fructose syrup, palm oil, monosodium glutamate; they mainly eat at home, treat lunch at a pizzeria or a takeaway as a nice variety or a so-called ‘emergency exit’. They like experimenting with food, e.g., they like to try different cuisines; they do not know about alternatives to sweets and snacks, they buy mainly from the mainstream offer; they like exercising and walking; they spend a lot of time with their families	they do not care what, when, and how they eat; they live on the run; they lack the habit of eating the right number of meals at regular times; they do not avoid fast food such as a hot dog at the petrol station or a kebab; they use semifinished and ready-made meals at home; are often overweight, spend little time outdoors, and have a very stationary lifestyle (car, home, and work)	eat fairly healthy, but it is not a priority; they believe that their child knows the required basics and that is enough for them; it is more important for them to learn, know, perform, and achieve for their child to have a better start in the future; busy, in a hurry, travel a lot and are away from home; they like to eat out and order takeaway food because it saves them time; they usually cook at weekends, which is quite satisfying. moderately active in sport (skiing in winter and occasional bike trips); buy quite a lot of premium products because they believe in their high quality
**A sense of responsibility to pass on knowledge and healthy habits to children:**			
high	high	low	medium
**Remaining restrictive on the subject of nutrition:**			
high level	high level	low level	low level
**Knowledge of proper nutrition:**			
high level	intermediate level	low level	low level

* The “distanced” profile was identified only in the group of parents of students aged 10–12.

## Data Availability

Data are contained within the article and Supplementary Materials.

## Supplementary Materials

The following supporting information can be downloaded at: https://www.mdpi.com/article/10.3390/nu15234930/s1, Supplementary file: Focus-Group Interview scenario.
