# Supplementary material for: The Family Environment as a Source for Creating the Dietary Attitudes of Primary School Students—A Focus Group Interview: The Junior-Edu-Żywienie (JEŻ) Project
Source: Nutrients. 2023 Nov 26;15(23):4930. doi: 10.3390/nu15234930 (PMC10708071; doi:10.3390/nu15234930)
Supplement: Supplementary file 1 [file nutrients-15-04930-s001.zip › nutrients-2716051-supplementary.pdf]

Ministerstwo Edukacji i Nauki

NARODOWY INSTYTUT ZDROWIA PUBLICZNEGO
PAŃSTWOWY INSTYTUT BADAWCZY

FOCUS GROUP INTERVIEW SCRIPT FOR PARENTS OF PRIMARY SCHOOL CHILDREN (AGED 7-9 AND 10-12 YEARS OLD)"

Participants of the interview: 6-8 parents of primary school children aged 7-9 and 10-12 years old
FGI duration: 90 minutes

**1. Meeting warm-up – introduction to the focus group (5 minutes)**
• Moderator's introduction, getting to know each other<br>• Presentation of the research and the group interviewing purpose<br>• Explanation of the rules for conducting the group interview<br>• Introducing the focus group to the fact that the discussion will be recorded, the recorded content is confidential and will be used only for research purposes<br>• Querying participants if they have any questions – moderator's responses and clarification of any doubts<br>• Informing about the duration of the meeting: up to 1.5 hours
**2. Evaluating children's parents' approach to nutrition (5 – 10 minutes)**
How important is the topic of proper nutrition, diet, valuable products, etc. in your homes/families?<br>We wait for spontaneous responses and then ask:<br>• Do you like these topics? Are you happy to discuss them?<br>• How important do you think they are?<br>• How often do you discuss these topics with your children?<br>• Are your children interested in these topics?<br>• From your point of view, are these topics easy to discuss?<br>*The moderator can ask the following questions on each topic:*<br>- Why yes/no?<br>- What is the reason for this?
**3. The current state of nutritional education for children in the opinion of parents (15-20 minutes)**
How do you assess the level of nutritional knowledge/awareness of your children (grades 1-6) in terms of:<br>• Rules/recommendations in terms of proper nutrition?<br>• Healthy and unhealthy food products?<br>• Methods of food production?<br>*The moderator can ask the following questions on each topic:*<br>• Why do you think so?<br>• What does your answer mean?<br>• What information do children particularly lack in this area?<br>Are these topics important from your point of view?<br>- Why do you think so?<br>- What does your answer result from?<br>Do you think that current curricula include content related to:<br>- Rules of proper nutrition?<br>- Food products?

„The task entitled 'Conducting scientific research in the field of nutrition of children and adolescents, developing and implementing a nutritional education program for pupils of grades 1-6 of primary schools. Acronym: Junior-Edu-Żywienie (JEŻ)'.
The study was financially supported by the Polish Ministry of Education and Science
(MEiN/2022/DPI/96 of March 7, 2022).

- Methods of food production?

If so, in which classes are these topics discussed the most often?

Do you think that food and nutrition topics should be included in teaching curricula?

- Why yes/no? Why you think so?
- What benefits do you see from discussing such topics at school?
- What weak points do you notice when discussing such topics at school?

- What specific areas related to nutrition do you think should be included in school curricula?
- What knowledge does your children need/lack? What problems do you struggle with in terms of your child's diet and nutrition?

The moderator collects spontaneous answers, and if they do not appear, also asks about:

- sweets, sugar, sweetened drinks;
- energy drinks;
- dairy products;
- whole grain cereal products;
- daily intake of vegetables and fruits;
- optimal number of meals per day;
- optimal time for meals eating during the day;
- portions' sizes and proportions of ingredients/product groups;
- liquid intake during the day;
- fast food;
- salted snacks;
- physical activity;
- nutrients, sources and roles of: fats, calcium, vitamins, fiber, protein, etc.
- vegan diet.

The moderator asks briefly:

- How important is this topic for you? Where it comes from?
- Why is this topic problematic in your home/in your children's nutrition?
- Which aspects are particularly important and therefore there should be more education about them?

4. Sources of information among students from the perspective of parents (10-15 minutes)

- Where do children get knowledge about topics that interest them? (we asked generally, to identify the sources most relevant to them)
  - Channels of famous youtubers: what specific names?
  - Influencers: what specific people, names, nicknames?
  - Facebook, Instagram: what specific profiles?
  - TikTok: what specific profiles?
  - Blogs: what specific names?
  - Peers?
  - Open and closed groups on social media: what topics and main thread?
  - Youth press: what titles?
  - Books and guides: what titles?
  - Teachers
  - Parents

*„The task entitled 'Conducting scientific research in the field of nutrition of children and adolescents, developing and implementing a nutritional education program for pupils of grades 1-6 of primary schools. Acronym: Junior-Edu-Żywienie (JEŻ)'.
The study was financially supported by the Polish Ministry of Education and Science
(MEiN/2022/DPI/96 of March 7, 2022).*

- TV programs: what specific names?

- What is the role of social media, influencers, YouTubers, and bloggers in students' acquisition of nutritional knowledge?
  - Do you think it can also be an important source of knowledge in the field of nutrition? Why is this possible (or impossible)?
  - What conditions would have to be fulfilled for these to be important sources of knowledge? Interesting content, appropriate intensity, attractive graphics?

5. The scope and implementation of nutritional education for children in the opinion of parents (10-15 minutes)

- What do you think nutritional education for children at school should look like?/How nutritional education for children at school should look like?
  - A separate teaching subject
  - Discussions during educational classes with class teacher
  - Extra teaching hours
  - Meeting with specialists: dietitians, doctors, athletes, etc.
- How long do you think nutritional education should be continued at school? (a few hours a year, a whole semester, several years)?
- Should nutritional education at school be differentiated depending on the age of children? What age groups do you propose to divide students into? What it comes from?
- Should classes/lessons be only theoretical, or should they also include practical issues? Which one, for example? What should children learn during such lessons?
- Who should be responsible for nutritional education at school? School director, class teacher, nurse, external institution, who else?

6. Scope and implementation of nutritional education for parents (5-10 minutes)

- Do you think that nutritional education for students' parents should be provided in primary school? If so, what methods would you like to suggest? What kinds of teaching forms would be attractive to you?
  - meeting with specialists: dietitians, doctors, athletes, etc.
  - cyclical lectures devoted to specific topics/problems
  - theoretical and practical classes for parents and children (together)
  - online form/offline
  - printed materials: folders, books, brochures, research results, exemplary diets and nutritional patterns
  - practical activities

7. Educational materials and parents' communication needs (10 minutes)

- What is needed to implement nutritional education? For example, availability of materials for parents and children, that increases the level of knowledge? More pressure from the school/ Ministry of National Education/ Ministry of Health?
- What media should be used in children's nutritional education? (TV, radio, social media)
- What publications addressed to children should be prepared as part of nutritional education (books, guides, comics, etc.)?
- What form of educational materials is the most beneficial?

„The task entitled 'Conducting scientific research in the field of nutrition of children and adolescents, developing and implementing a nutritional education program for pupils of grades 1-6 of primary schools. Acronym: Junior-Edu-Żywienie (JEŻ)'.
The study was financially supported by the Polish Ministry of Education and Science
(MEiN/2022/DPI/96 of March 7, 2022).

- printed
- multimedia
- video films etc.

- What additional teaching aids should be prepared for nutritional education to be effective? Do you have any specific ideas?
- Should social media be used in nutritional education and how? Do you have any specific ideas?

8. The pandemic and dietary habits (5 minutes)

- Do you still remember the times of the pandemic? Your children couldn't go to school, and education was provided mainly online, so you probably spent most of your time at home with children.
- What particularly changed in your life then?
- How did your daily routine change during that time? What changes occurred?
- How did you feel?
- How did you spend your time?
- Did you engage in any additional activities?
- Do you feel that the whole family had more or less physical activity?
- Do you feel you ate different foods or meals than usual during that time? What foods do you associate with that period?
- What types of foods did you eat more? Do you still eat those products? Why yes/no?
- What types of foods did you eat seldom?
- Do you feel you are eating in the same way as during the pandemic?

9. Summary of the group discussion (5 minutes)

- A brief summary of the discussion.
- Is there anything else anyone would like to add?
- Thank you for participating in the interview.

„The task entitled 'Conducting scientific research in the field of nutrition of children and adolescents, developing and implementing a nutritional education program for pupils of grades 1-6 of primary schools. Acronym: Junior-Edu-Żywienie (JEŻ)'.
The study was financially supported by the Polish Ministry of Education and Science
(MEiN/2022/DPI/96 of March 7, 2022).
